# Transcriptome Analysis Reveals Potential Regulators of DMI Fungicide Resistance in the Citrus Postharvest Pathogen *Penicillium digitatum*

**DOI:** 10.3390/jof10050360

**Published:** 2024-05-18

**Authors:** Yue Xi, Jing Zhang, Botao Fan, Miaomiao Sun, Wenqian Cao, Xiaotian Liu, Yunpeng Gai, Chenjia Shen, Huizhong Wang, Mingshuang Wang

**Affiliations:** 1 College of Life and Environmental Sciences, Hangzhou Normal University, Hangzhou 311121, China; xyue675@163.com (Y.X.); zhangjing6@stu.hznu.edu.cn (J.Z.); hualuooo@outlook.com (B.F.); 2022210304040@stu.hznu.edu.cn (M.S.); caowenqian2024@163.com (W.C.); 2021210315139@stu.hznu.edu.cn (X.L.); shencj@hznu.edu.cn (C.S.); whz62@163.com (H.W.); 2 School of Grassland Science, Beijing Forestry University, Beijing 100083, China; gaiyunpeng@bjfu.edu.cn

**Keywords:** *Penicillium digitatum*, fungicide resistance, ergosterol biosynthesis, transcription factor, *flbC*

## Abstract

Green mold, caused by *Penicillium digitatum*, is the major cause of citrus postharvest decay. Currently, the application of sterol demethylation inhibitor (DMI) fungicide is one of the main control measures to prevent green mold. However, the fungicide-resistance problem in the pathogen *P. digitatum* is growing. The regulatory mechanism of DMI fungicide resistance in *P. digitatum* is poorly understood. Here, we first performed transcriptomic analysis of the *P. digitatum* strain Pdw03 treated with imazalil (IMZ) for 2 and 12 h. A total of 1338 genes were up-regulated and 1635 were down-regulated under IMZ treatment for 2 h compared to control while 1700 were up-regulated and 1661 down-regulated under IMZ treatment for 12 h. The expression of about half of the genes in the ergosterol biosynthesis pathway was affected during IMZ stress. Further analysis identified that 84 of 320 transcription factors (TFs) were differentially expressed at both conditions, making them potential regulators in DMI resistance. To confirm their roles, three differentially expressed TFs were selected to generate disruption mutants using the CRISPR/Cas9 technology. The results showed that two of them had no response to IMZ stress while ∆*PdflbC* was more sensitive compared with the wild type. However, disruption of *PdflbC* did not affect the ergosterol content. The defect in IMZ sensitivity of ∆*PdflbC* was restored by genetic complementation of the mutant with a functional copy of *PdflbC*. Taken together, our results offer a rich source of information to identify novel regulators in DMI resistance.

## 1. Introduction

Citrus (including all species in the *Citrus* genus) is one of the most important economic crops in the world, with its fruit yield ranking first. However, postharvest diseases caused by microbial pathogens during packaging, storage, transportation, and marketing can cause heavy economic losses of up to 30–50% of the total production when fruits are not timely treated with postharvest fungicides and/or placed in refrigeration [1]. Citrus green mold caused by *Penicillium digitatum* is the main cause of postharvest disease in citrus, which accounts for up to 90% of the total postharvest losses [2]. At present, the massive application of fungicides to marketed fruits is the major means to control the citrus green mold. In the mid-1970s, the sterol demethylation inhibitor (DMI) fungicides imazalil (IMZ) and prochloraz were introduced to control postharvest diseases of citrus fruits worldwide, owing to their good inhibitory effects on fruit decay symptoms, and these fungicides are still widely used in citrus preservation all over the world [3]. However, with the extensive and continuous use of DMI fungicides, the resistance problem in pathogens including *P. digitatum* is growing. According to previous studies, the resistance of pathogenic fungi to DMI fungicides is mainly caused by three molecular mechanisms: (a) point mutation occurs in the coding region of target gene *CYP51s*, and the resultant 14α-demethylase cannot bind to drugs or has a decreased drug affinity [4,5,6,7,8], (b) the target gene *CYP51s* is overexpressed, resulting in the need for increased drug dose to inhibit the activity of 14α-demethylase [5,9,10,11,12,13], or (c) the expression of ATP-binding cassette (ABC) transporters or major facilitator superfamily (MFS) transporters on the cell membrane was up-regulated, which leads to accelerated elimination of intracellular drugs [14,15]. The reported molecular mechanisms of DMI resistance in *P. digitatum* are mainly caused by overexpression of drug target genes, which can be divided into R1, R2, and R3 resistance types: the presence of five tandem repeats of a 126-bp enhancer in the *CYP51A* gene’s promoter (R1) [16], the insertion of a unique 199-bp transposable element in the *CYP51A* gene’s promoter (R2) [17], and the insertion of the 199-bp element in the *CYP51B* gene’s promoter (R3) [18]. The R3 resistance type now exists predominantly in the DMI-resistant population of *P. digitatum* in the main producing areas of citrus, making the control strategies severely challenged [18,19].

As the ergosterol synthesis pathway is targeted by DMI fungicides, how this pathway is regulated has received widespread attention. In eukaryotes, the primary regulators of the sterol production pathway are called sterol regulatory element binding proteins, or SREBPs [20]. SREBPs belong to the bHLH class of transcription factors and are widely found in fungi from genera like *Cryptococcus*, *Schizomyces*, and *Aspergillus* [21]. SREBPs can recognize and bind to sterol regulatory elements (SREs) in the promoter region of sterol synthesis pathway genes, thereby regulating the expression of target genes [22]. However, the copy number and function of SREBP homologous protein in different species may differ. For example, two SREBP homologs, Sre1 and Sre2, are found in *Schizosaccharides pombe*; Sre1 is the main regulator of ergosterol synthesis and hypoxic adaptation, while Sre2 is not involved in the regulation of these two important functions [23]. In *Aspergillus fumigatus*, two SREBP homologous proteins, SrbA and SrbB, have been identified, and both show an important role in hypoxic growth and pathogenicity [24]. In *Magnaporthe oryzae*, the SREBP encoding gene deletion mutant, Δ*Mosre1*, showed increased susceptibility to hypoxia and delayed growth in host cells. However, such defects did not result in a substantial decrease in disease severity [25]. In the medicinal fungus *Ganoderma lingzhi*, the metabolism of Ganoderic acids, lipids, and ergosterol was significantly increased when SREBP was overexpressed [26]. There are two SREBP homologous proteins, PdSreA and PdSreB, in *P. digitatum*. Deletion of *PdsreA* or *PdsreB* led to abnormal mycelia growth, decreased intracellular ergosterol content, and increased susceptibility to DMI fungicide, but the influence on virulence is very limited [27].

Although some yeast fungi like *S. cerevisiae*, *C. albicans*, *C. glabrata*, and *C. parapsilosis* also contain SREBP homologous proteins, these SREBPs are not involved in the regulation of intracellular ergosterol synthesis [21]. Instead, these fungi utilize transcription factor Upc2 to control ergosterol synthesis, drug resistance, and hypoxic response [28,29,30,31,32]. Surprisingly, both SREBP and Upc2 exist in the *Fusarium graminearum* genome; however, they are not associated with ergosterol regulation and sensitivity to DMI fungicides. Intriguingly, a transcriptional factor named FgSR in *F. graminearum* was reported to bind to the promoter region of 20 genes involved in sterol synthesis, and deletion of the *FgSR* gene resulted in decreased sterol synthesis, increased DMI sensitivity, less deoxynivalenol production, and reduced virulence, proving that FgSR is a novel and master regulator of sterol biosynthesis in *F. graminearum* [33].

According to previous studies, it is speculated that there may be a variety of regulatory pathways controlling ergosterol synthesis in fungi. In addition to SREBPs, other transcription factors that may participate in the regulation of ergosterol content and DMI resistance in *P. digitatum* are still not known. Thus, the objective of this study is to find novel regulators of DMI fungicide resistance in the citrus postharvest pathogen *P. digitatum*.

## 2. Materials and Methods

### 2.1. Fungal Strains and Growth Conditions

This study was conducted with the wild-type *P. digitatum* strain Pdw03 (CBS130527), isolated from an infected citrus fruit (Quzhou of Zhejiang province, China) and resistant to IMZ [34]. Pdw03 and its derived mutants were kept on solid potato dextrose agar (PDA), and conidia were collected after 5–7 days of incubation. Mycelia were harvested from potato dextrose broth (PDB) after incubation at 160 rpm at 25 °C for 2 days. To prepare RNA sequencing samples, conidial suspensions (10^6^ spores/mL) 100 μL of *P. digitatum* Pdw03 were cultured in PDB medium at 25 °C for 48 h, and IMZ was then added to PDB medium (6 μg/mL), and samples were collected at 0, 2, and 12 h, respectively.

### 2.2. Transcriptome Analysis

Each sample’s collected mycelia were ground in liquid nitrogen, and total RNA was extracted with an RNAprep Pure Kit (Tiangen Biotech Co., Ltd., Beijing, China). RNA-Seq libraries were generated with the Illumina TruSeq RNA Sample Preparation Kit and sequenced on an Illumina NovaSeq6000 platform by the Biomarker Technology Co., Ltd. (Beijing, China). The RNA-Seq was conducted for three biological replicates of each sample. Trimmomatic v0.32 was used to filter low-quality reads and remove adaptor sequences from raw reads [35]. Clean reads were mapped to the reference genome with HISAT2 [36]. The number of reads mapped to each gene was counted using featureCounts v2.0.0 [37]. Principal component analysis (PCA) was performed to identify whether samples clustered based on treatment using the function prcomp() in R 4.2.3 (https://rdrr.io/r/stats/prcomp.html (accessed on 10 March 2023)). The differential gene expression was analyzed using DESeq2 [38]. Transcripts were considered differentially expressed when their absolute value of log2 fold change (log2FC) was more than 1 and the false-discovery rate (FDR) value was less than 0.01. DEGs were analyzed for gene ontology (GO) enrichment using topGO v2.28.0 [39]. Kyoto Encyclopedia of Genes and Genomes (KEGG) pathway analysis was conducted with KOBAS-I [40]. The transcriptome project has been deposited at NCBI BioProject under the accession PRJNA1072022.

### 2.3. Quantitative Real-Time PCR

To validate the reliability of the transcriptome data, we chose 8 genes to measure their expression levels through qRT-PCR. Reverse transcription was performed with 1 μg of each RNA sample using the HiScript III All-in-one RT SuperMix reagent kit (Vazyme Biotech Co., Ltd., Nanjing, China). The target genes’ relative transcript levels were determined in triplicate using a 7500 Real-Time PCR equipment (ABI, Foster City, CA, USA). The actin gene (AB030227) served as an internal reference, and the data were analyzed using the 2^−ΔΔCT^ method.

### 2.4. Disruption of P. digitatum Genes and Mutant Complementation

To ensure their roles in DMI resistance, we then selected 3 differentially expressed transcription factor encoding genes, *Pdw03_2654* (bZIP, down-regulated), *Pdw03_3762* (Zn_clus, down-regulated), and *Pdw03_3571* (zf-C2H2, up-regulated, *PdflbC*), for functional analyses in *P. digitatum*. Gene disruption was performed according to the recently developed CRISPR/Cas9 tools in *P. digitatum* with minor modification [41]. The 20-bp protospacer for each target gene was designed with the GuideMaker v0.4.0 software [42]. The sgRNA expression constructs were synthesized by Shangya Biotechnology Co., Ltd., Hangzhou, China. After digesting with the *Bgl*II ligase, the sgRNA expression construct was inserted into the pFC332 vector to generate the gene disruption plasmid pFC332-Gene-D. Then, this vector was transformed into the protoplasts of *P. digitatum* Pdw03 according to the previously described protocol [41]. Transformants grown on PDA with hygromycin (100 mg/L) were isolated, and mutants were confirmed by PCR and Sanger sequencing. For genetic complementation, a functional copy of *PdflbC* DNA fragment under its native promoter was amplified and inserted into the pA1300-NEO vector [43]. The resultant vector was subsequently transformed into ∆*PdflbC* protoplasts. Transformants were recovered from neomycin-supplemented PDA (100 mg/L) and examined by PCR and Sanger sequencing. Primers are listed in Table S1.

### 2.5. Mycelial Growth Tests

To know whether these transcription factors play a role in environmental adaptation, *P. digitatum* wild type and mutant strains were grown on PDA plates containing 4 μg/mL IMZ, 0.7 M NaCl, 0.05 mM Menadione, or 200 μg/mL Congo red (CR), 200 μg/mL Sodium dodecyl sulfate (SDS). Each plate was inoculated with a 5 mm mycelial plug, and the colony diameters were measured after 5 days of incubation at 25 °C. The percentage of the mycelial radial growth inhibition (MRGI) rate was calculated using the formula MRGI% = ((C − N)**/**(C − 0.5)) × 100%, where C is colony diameter of the control (PDA only) and N is that of a treatment. The experiments were repeated thrice.

### 2.6. Virulence Assays

To examine the fungal virulence, infection assays were performed on mandarin (*Citrus reticulata* Blanco) fruits using the wild type and 3 gene deletion mutants of *P. digitatum*. Citrus peel was first wounded (1–2 mm deep) with sterile needles and then inoculated with 10 μL conidial suspensions (1 × 10^6^ conidia/mL) of the wild type and mutant strains of *P. digitatum*. The infected citrus fruits were kept at 25 °C for 4 days before being tested for lesion size. Six fruits were inoculated for each strain, and the experiment was conducted two times.

### 2.7. Determination of Intracellular Ergosterol Content

To extract ergosterol, fungal spores were placed in a 50 mL yeast glucose (YG) medium (15 g/L glucose, 5 g/L yeast extract), which was then shaken continuously at 25 °C for two days. Mycelia were then collected for ergosterol extraction according to the published protocol (Wang et al., 2014 [44]). Each sample was analyzed using the Waters e2695 high-performance liquid chromatography (HPLC) system. Ergosterol was separated at room temperature on a Discovery C18 250 nm × 4.6 nm, 5 μL analytical column using 100% methanol (chromatography pure) as mobile phase. Quantification of ergosterol was measured based on retention time and co-chromatography of a commercial standard of ergosterol (Sigma, St. Louis, MO, USA) under the detection wavelength of 282 nm. This experiment was repeated twice.

## 3. Results

### 3.1. Transcriptomic Analysis of P. digitatum in Response to IMZ

**Figure 1:**
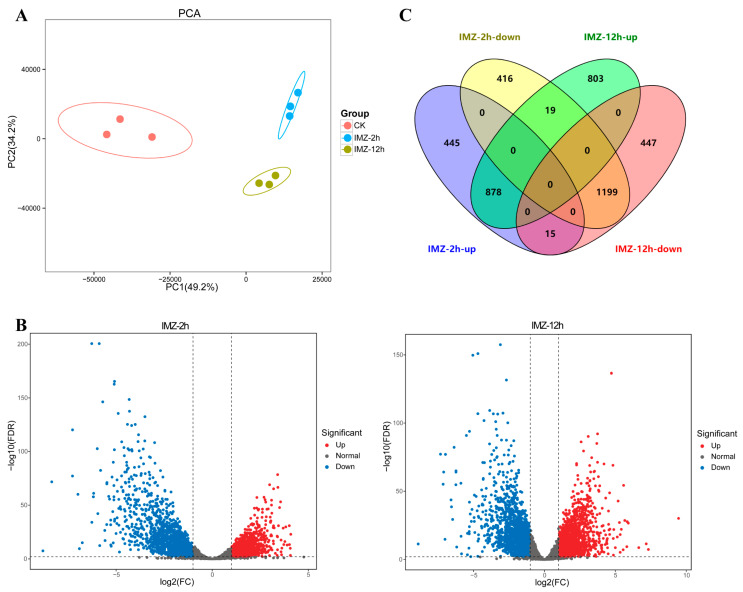
Overview of the differentially expressed genes. (**A**) PCA plot of RNA-sequencing samples. Each dot represents a sample. The distance between dots represents the similarity of samples. The horizontal axis and the vertical axis represent the contribution of PC1 and PC2 to the identification of distinct samples. (**B**) Volcano plots of global gene expression pattern. Each dot represents a gene. Blue dots are down-regulated genes, while red dots are up-regulated ones, and black dots are genes without significant difference. (**C**) Venn diagram displaying the up-regulated and down-regulated genes after IMZ treatment for 2 h or 12 h compared to that of the control 0 h.

The number of clean reads ranged from 20,838,987 to 29,875,237, and the percentage of Q30 bases in all samples was no less than 89% (Table S2). The proportion of reads that aligned to the genome ranged between 86.9 and 93.8% (Table S2). As demonstrated in Figure 1, principal component 1 (PC1) accounts for 49.2% of the sample variability, whereas principal component 2 (PC2) accounts for 34.2%. Each condition was clearly distinguished from the other, and samples in the same condition were more similar (Figure 1A). These results indicate the good quality of the transcriptome data.

**Figure 2:**
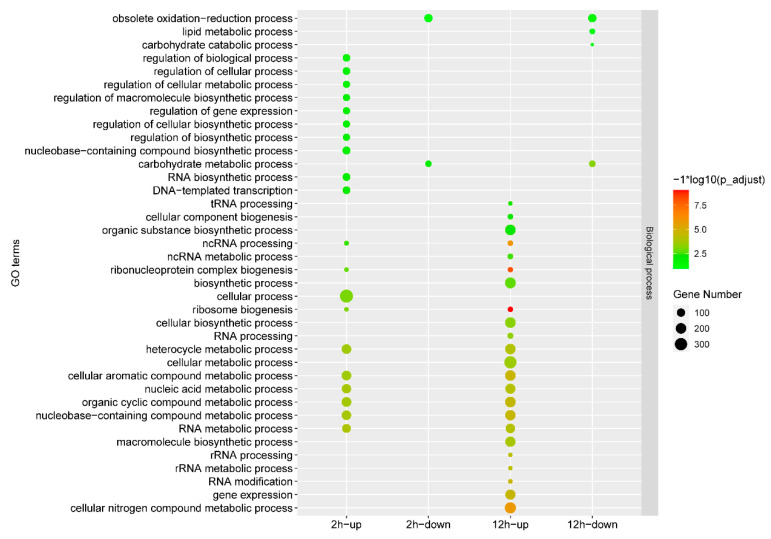
GO analysis of the differentially expressed genes under different conditions. The *x*-axis indicates different gene sets. The *y*-axis indicates the GO term.

**Figure 3:**
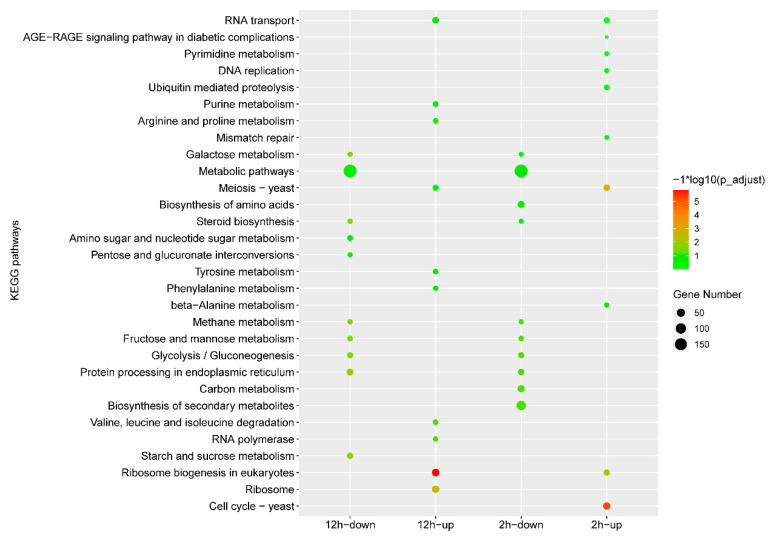
KEGG analysis of the differentially expressed genes under different conditions. The *x*-axis indicates different gene sets. The *y*-axis indicates the pathway name.

Gene expression comparisons were then performed between the IMZ treatment and control samples, i.e., 2 h vs. 0 h and 12 h vs. 0 h. In total, 2973 differentially expressed genes, including 1338 up-regulated genes and 1635 down-regulated genes, were identified in *P. digitatum* under IMZ treatment for 2 h compared to the control (Figure 1B,C). GO enrichment analysis revealed that the up-regulated genes were significantly enriched for the biological progress categories “nucleic acid metabolic process”, “RNA metabolic process”, “organic cyclic compound metabolic process”, “nucleobase-containing compound metabolic process”, “cellular aromatic compound metabolic process”, “heterocycle metabolic process”, “ribosome biogenesis”, “cellular process”, “ribonucleoprotein complex biogenesis”, “ncRNA processing”, and “DNA-templated transcription”. Nevertheless, GO analyses did not show any significant annotation in the down-regulated gene set (Figure 2). KEGG analysis showed that the up-regulated genes were significantly enriched in “Cell cycle”, “Meiosis “, and “Ribosome biogenesis in eukaryotes”. However, the down-regulated genes were not significantly enriched in any pathway (Figure 3).

Overall, 3361 genes were differentially expressed in *P. digitatum* under IMZ treatment for 12 h compared to control, comprising 1700 up-regulated and 1661 down-regulated genes (Figure 1B,C). GO enrichment analysis revealed that the up-regulated genes were significantly enriched for the biological progress categories “ribosome biogenesis”, “ribonucleoprotein complex biogenesis”, “cellular nitrogen compound metabolic process”, “cellular aromatic compound metabolic process”, “gene expression”, “organic cyclic compound metabolic process”, “nucleobase-containing compound metabolic process”, “nucleic acid metabolic process”, “heterocycle metabolic process”, “macromolecule biosynthetic process”, “cellular metabolic process”, “cellular biosynthetic process”, “organic substance biosynthetic process”, “cellular component biogenesis”, and some RNA related categories such as “RNA modification“ and “RNA processing”, while those down-regulated genes were only significantly enriched for the category “carbohydrate metabolic process” (Figure 2). KEGG pathway enrichment analysis showed the up-regulated genes were significantly enriched in “Ribosome” and “Ribosome biogenesis in eukaryotes”, while the down-regulated genes were significantly enriched in “Starch and sucrose metabolism”, “Protein processing in endoplasmic reticulum”, “Glycolysis/Gluconeogenesis”, “Methane metabolism”, “Fructose and mannose metabolism”, “Steroid biosynthesis”, and “Galactose metabolism” (Figure 3).

The results of qRT-PCR revealed that though the level of fold changes between the two techniques for several of the genes in the two conditions varied, both generally displayed comparable patterns in transcript accumulation (Figure S1).

### 3.2. Expression of the Ergosterol Biosynthesis Pathway

**Figure 4:**
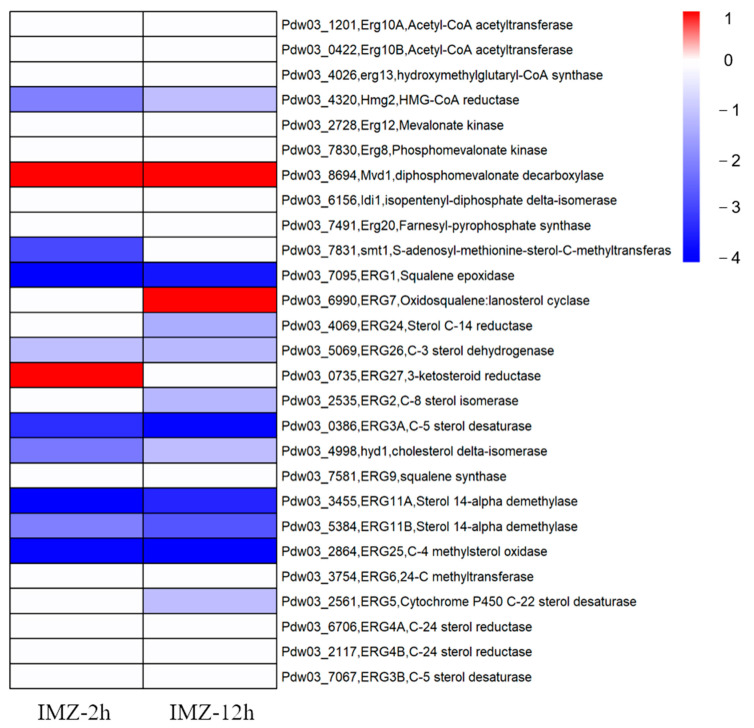
Heat map visualization of gene expressions associated with the ergosterol biosynthesis pathway under different conditions.

The results showed that the expression of about half of the genes in this pathway was affected after IMZ treatment. All DEGs involved in ergosterol biosynthesis were down-regulated except for three genes encoding Mvd1 (diphosphomevalonate decarboxylase), ERG7 (lanosterol cyclase), and ERG27 (3-ketosteroid reductase), which up-regulated their expression level by 2~3 folds. Reduced expression of the genes encoding Hmg2 (HMG-CoA reductase), ERG1 (Squalene epoxidase), ERG26 (C-3 sterol dehydrogenase), ERG3A (C-5 sterol desaturase), Hyd1 (cholesterol delta-isomerase), ERG25 (C-4 methylsterol oxidase), and ERG11A/B (Sterol 14-alpha demethylase) was detected in both conditions. The transcript levels of left DEGs, namely smt1, ERG24, ERG2, and ERG5, were significantly decreased in only one condition (2 h or 12 h post IMZ treatment) (Figure 4).

### 3.3. Expression of Potential Regulators

After comparing two sets of DEGs associated with IMZ treatment, 878 and 1199 DEGs were up-regulated and down-regulated in both conditions, respectively (Figure 1C). We then searched for potential regulators of ergosterol biosynthesis and DMI resistance from these core DEGs. Finally, a total of 84 transcription factors were discovered to be differentially expressed (26.3% of all transcription factors in the genome), with the expressions of 54 being up-regulated and 30 being down-regulated (Table S3).

### 3.4. Disruption of Three Transcription Factors in P. digitatum

**Figure 5:**
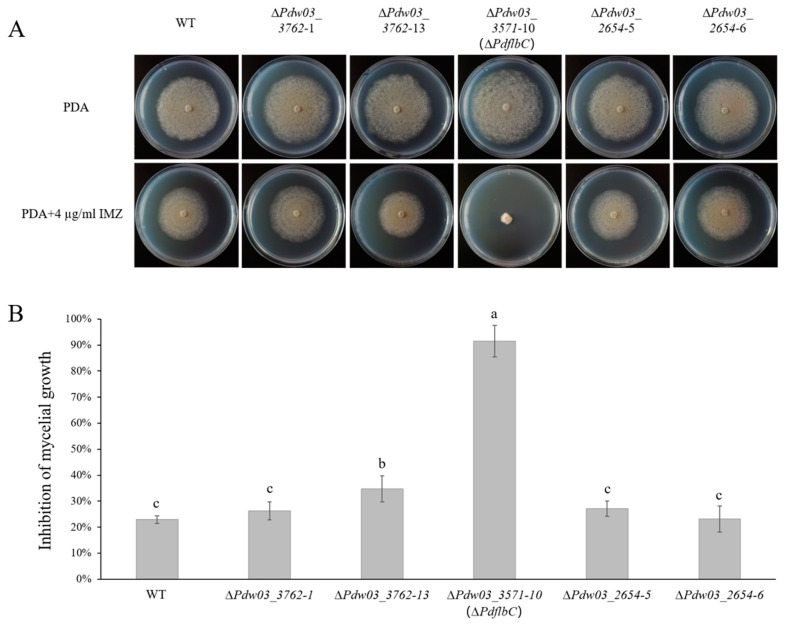
Sensitivity of the wild type and gene deletion mutants to IMZ. (**A**) Colony morphology of the wild type and gene deletion mutants grown on potato dextrose agar (PDA) supplemented with or without IMZ. All plants were incubated at 25 °C for 5 days. (**B**) Growth inhibition of gene deletion mutants grown on PDA supplemented with or without IMZ. Different lowercase letters indicate significant difference estimated by Duncan’s test (*p* ≤ 0.05).

We obtained two mutants for *Pdw03_2654*, two for *Pdw03_3762*, and one for *Pdw03_3571* (Figure S2). The growth assay showed that the average colony diameter of the ∆*PdflbC* mutant was slightly larger than that of the wild type, while the others showed a similar growth rate with the wild type (Figure 5). However, the vegetative growth of ∆*PdflbC* was strongly retarded on medium supplemented with 4 μg/mL IMZ fungicide, while the other mutants and the complemented CP*PdflbC* mutant showed wild-type levels of growth (Figure 5 and Figure S3A). These results demonstrated that *PdflbC* is a crucial regulator in DMI resistance in *P. digitatum*.

**Figure 6:**
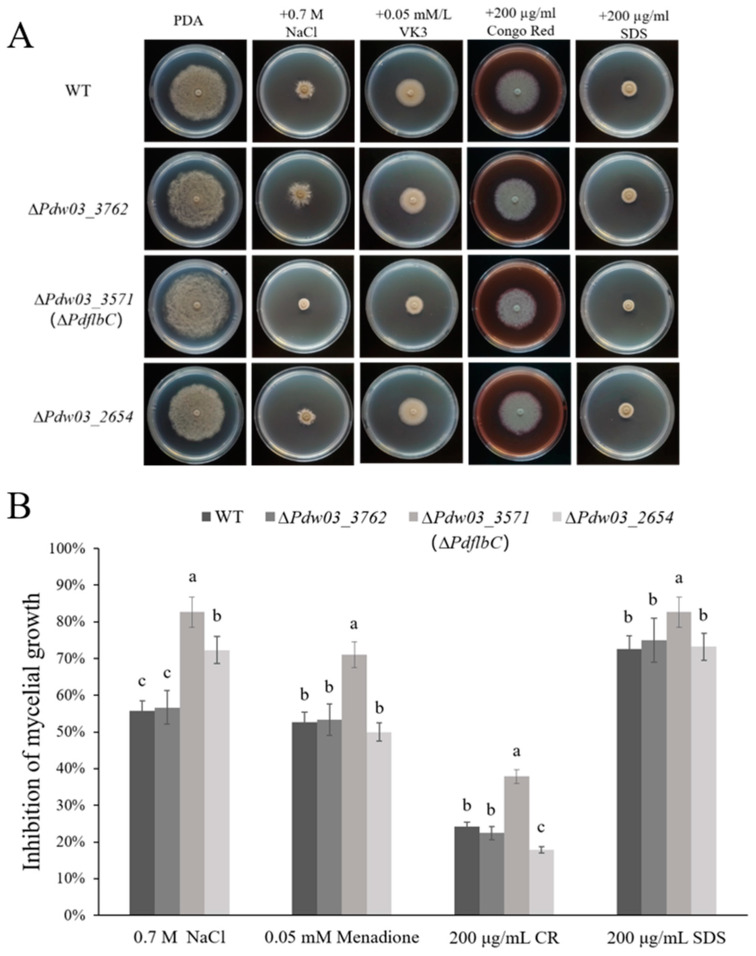
Sensitivity of the wild type and gene deletion mutants to environmental stresses. (**A**) Mycelial plugs of different strains were placed on PDA supplemented with NaCl, Menadione, Congo red (CR), and Sodium dodecyl sulfate (SDS) at the concentrations indicated in the figure. All plates were incubated at 25 °C for 5 days. (**B**) Growth inhibition of gene deletion mutants grown on PDA supplemented with different chemicals. Different lowercase letters indicate significant difference estimated by Duncan’s test (*p* ≤ 0.05).

The stress assays showed that ∆*Pdw03_3762* exhibited similar phenotypes to the wild-type strain, ∆*Pdw03_2654* was more sensitive to salt stress, and the ∆*PdflbC* mutant exhibited significantly increased sensitivity to osmotic stress, menadione, and cell wall interfering agents CR and SDS (Figure 6). All phenotypic defects of ∆*PdflbC* excluding the elevated sensitivity to NaCl were restored by genetic complementation of the mutant with the wild-type *PdflbC* (Figure S3B). These results indicated that *PdflbC* is essential for the adaptation to environmental stresses in *P. digitatum*.

**Figure 7:**
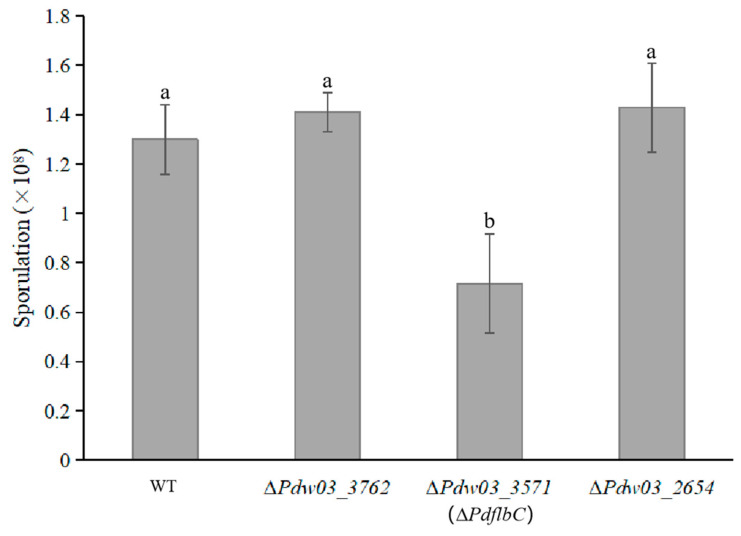
Comparison of sporulation of the wild type and gene deletion mutants. Strains were inoculated on PDA at 25 °C for 5 days, and conidia were then collected and counted in a hemocytometer. Different lowercase letters indicate significant difference estimated by Duncan’s test (*p* ≤ 0.05).

Both ∆*Pdw03_3762* and ∆*Pdw03_2654* generated slightly increased spores compared with the wild-type strain, while ∆*PdflbC* produced only half the spores of the wild type (Figure 7). The wild-type sporulation level was restored in CP*PdflbC* (Figure S3C). However, deletion of these three genes did not affect germination time and rate of conidia compared with WT.

**Figure 8:**
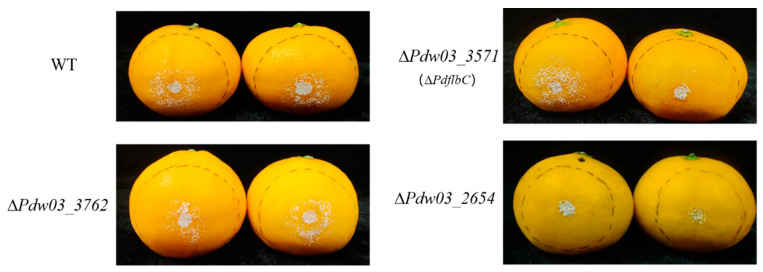
Decay symptoms of the wile type and gene deletion mutants on citrus fruits. Mandarin (*Citrus reticulata* Blanco) fruits inoculated with the indicated strains were incubated at 25 °C for 4 days.

The virulence assay revealed that all gene deletion mutants caused similar maceration lesions with the wild type, suggesting that these genes are dispensable for the pathogenicity of *P. digitatum* (Figure 8).

### 3.5. Roles of PdflbC in Regulating Ergosterol Biosynthesis

**Figure 9:**
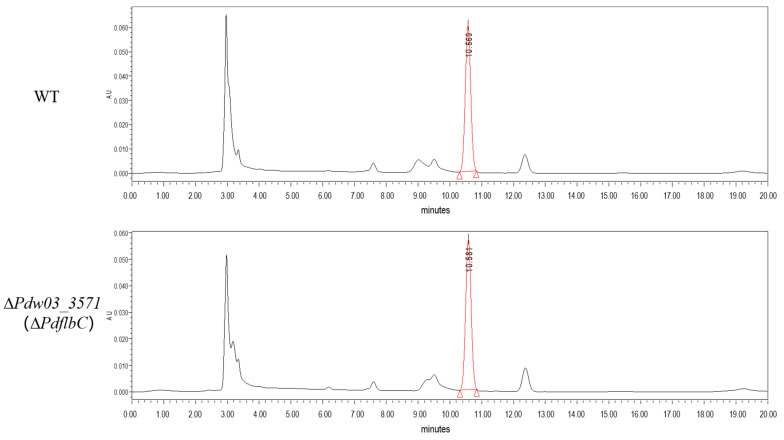
High-performance liquid chromatography (HPLC) analysis of ergosterol content from the wild type and the ∆*PdflbC* mutant.

Ergosterol content of the wild type and the ∆*PdflbC* mutant were quantified using the HPLC method, and the results showed that no significant differences were found between them (Figure 9).

## 4. Discussion

*P. digitatum* is the most common cause of postharvest decay in citrus fruits around the world. At present, the problem of DMI fungicide resistance in the *P. digitatum* population is rising due to the extensive and continuous use of fungicides for decades. Several mechanisms underlying DMI resistance have been documented, such as enhanced gene expression because of insertions in the promoter or alterations in the coding area [3,18,45]. The regulation of DMI fungicide resistance can be mediated by many regulators in fungi; nevertheless, related studies were poorly reported in *P. digitatum*. To gain further insight into the regulatory mechanism of DMI fungicide resistance in *P. digitatum*, we performed transcriptomic analysis of the *P. digitatum* strain Pdw03 before and after IMZ treatment at different time points and we showed how this strain responded to IMZ stress and identified potential regulators in this process; we also characterized the functions of three candidate genes using the CRISPR/Cas9 technology to show their relationship with IMZ sensitivity.

Our RNA-Seq data of the *P*. *digitatum* Pdw03 strain after being treated with IMZ for 2 and 12 h identified 2973 and 3361 differentially expressed genes, respectively (Figure 1). Function annotation analysis revealed that *P. digitatum* showed many similar responses to these two treatment times. The genes expressed at a higher degree were enriched in ribosome-related biological processes, such as ribonucleoprotein complex biogenesis, ribosome biogenesis, and nucleic acid metabolism, while the down-regulated genes were more related to carbohydrate metabolism like starch/sucrose metabolism and gluconeogenesis (Figure 2 and Figure 3). Previously, whole transcriptome analysis of *P. digitatum* HS-F6 strain treated with prochloraz was performed, and 1100 DEGs were identified after prochloraz treatment, with 402 up-regulated and 698 down-regulated. The top five functional categories of the DEGs of HS-F6 before and after prochloraz treatment included cation binding, ion binding, transition metal ion binding, metal ion binding, and tetrapyrrole binding [46]. These results revealed that, although both prochloraz and IMZ belong to DMI fungicide, their respective resistant strain showed different response patterns when fungicides were applied. This inconsistency can be explained by the phenomenon that the IMZ-resistant strain Pdw03 was more sensitive to prochloraz [27]. However, the underlying mechanism is not clear so far.

As the biosynthesis of ergosterol was mainly targeted by the DMI fungicides, it is expected that the expression of genes in the ergosterol biosynthesis pathway was significantly changed after DMI treatment. Usually, sterol biosynthetic genes up-regulated their expression after a DMI fungicide treatment, as maintenance or reinforcement of cell membrane integrity is a universal response when exposed to DMI fungicide. In *C. beticola*, 18 genes in the ergosterol biosynthesis pathway were induced in DMI-resistant strain after tetraconazole exposure [47]. Both *P. italicum* and *P. digitatum* showed overexpression of *CYP51* genes when treated with prochloraz [46,48]. However, in our transcriptome data, 12 ergosterol biosynthetic genes were underexpressed after IMZ treatment, while only 3 were overexpressed (Figure 4). This may be caused by the large dose of fungicide used in our experiment, and the vegetative growth of *P. digitatum* Pdw03 might be strongly inhibited under the concentration of 6 μg/mL used in this study.

Previous studies showed that transcription factors, such as SREBP, UPC2, and SR, are critical regulators of ergosterol synthesis in fungi [24,31,33]. In *P. digitatum*, two SREBP homologous proteins, PdSreA and PdSreB, were previously reported to mediate ergosterol biosynthesis and DMI resistance by regulating the expression of many related genes, for example, *Erg5*, *Erg10B*, *Erg7*, *Erg13*, *Erg3B*, *Erg1*, and *Cyp51A*/*Erg11A* [27]. To find more regulators in DMI resistance, we identified 54 up-regulated and 30 down-regulated transcription factors, which might be good candidates for functional analyses (Table S3). Of them, functions of two transcription factors have been characterized in fungi. One is the *flbC* gene, which is crucial for the activation of conidiation and the co-ordination of vegetative growth and development in *A. nidulans* [49], and the other is the pH-responsive transcription factor PacC, which is essential for pH sensing and pathogenicity in some plant pathogenic fungi [50,51]. However, the functions of all these genes in mediating ergosterol content and DMI resistance are largely unknown, and whether they can play as critical regulators needs further investigation.

To help establish a role in DMI resistance, three genes were selected for functional characterization based on their expression levels. To our surprise, disruption of the conidiation-related transcription factor PdflbC led to markedly increased sensitivity of the mutant to IMZ (Figure 5). FlbC contains two C_2_H_2_ zinc fingers at the C-terminus and a putative activation domain at the N-terminus [52]. In *A. nidulans*, the lack of *flbC* causes delayed and reduced conidiation, *brlA* and *vosA* expression, and conidial germination, while overexpression of *flbC* inhibits radical growth and activates expression of *brlA*, abaA, and vosA [49]. In *P. oxalicum*, the deletion of *PoFlbC* led to reduced and delayed conidiation and down-regulated the expression of *brlA*, while overexpression of *flbC* slightly affected conidiation, impaired vegetative growth, and decreased the *brlA* expression [53]. In addition, the synthesis of cellulase and hemicellulase was significantly decreased after *PoflbC* gene deletion. Down-regulation of genes producing cellulases, hemicellulases, and other proteins with roles in lignocellulose breakdown was discovered in the *PoflbC* deletion mutant [53]. In *P. digitatum*, the *PdflbC* gene deletion mutant also showed reduced conidiation (Figure 7), indicating that *flbC* is a conserved upstream developmental activator in *Penicillium* and *Aspergillus*. However, whether *flbC* is required for DMI fungicide resistance in other fungi needs further investigation. Although the *PdflbC* gene is essential for DMI resistance, the ergosterol content of the *PdflbC* gene deletion mutant was not changed compared with that of the wild type (Figure 9), suggesting that *PdflbC*-mediated DMI fungicide resistance is in a way irrelevant to the ergosterol biosynthesis pathway, and the underlying mechanism remains elusive.

## 5. Conclusions

In this study, we performed transcriptomic analysis of the *P. digitatum* strain Pdw03 after IMZ treatment and identified a large number of differentially expressed genes. Functional analysis demonstrated that one transcriptional factor, *PdflbC*, is crucial for the tolerance of *P. digitatum* to IMZ. However, functions of the majority of candidate genes are largely unknown, and their roles in DMI resistance need further validation. Taken together, our results provide new insights into the mode of action of IMZ response in *P. digitatum* and offer a rich source of information to identify novel regulators in DMI resistance.

## Data Availability

The original contributions presented in the study are included in the article/Supplementary Material, further inquiries can be directed to the corresponding author.

## Supplementary Materials

The following supporting information can be downloaded at: https://www.mdpi.com/article/10.3390/jof10050360/s1, Figure S1. qRT-PCR validation of the RNA-Seq data. Figure S2. Sequence alignment of gene locus in the selected mutants. Protospacers were marked in red. Protospacer-adjacent motifs (PAM) were underlined. Nucleotide alterations are highlighted in yellow. Figure S3. Phenotypes of the complemented strain CP*PdflbC*. (A) Sensitivity of the wild type, the gene deletion mutant ∆*PdflbC* and the complemented strain CP*PdflbC* to IMZ. (B) Sensitivity of the wild type, the gene deletion mutant ∆*PdflbC* and the complemented strain CP*PdflbC* to environmental stresses. (C) Sporulation of the wild type and the complemented strain CP*PdflbC*. Table S1. Primers used in this study. Table S2. Statistics of sequencing data. Table S3. Differentially expressed transcription factors after IMZ treatment.
