# Supplementary material for: Transcriptome Analysis Reveals Potential Regulators of DMI Fungicide Resistance in the Citrus Postharvest Pathogen Penicillium digitatum
Source: J Fungi (Basel). 2024 May 18;10(5):360. doi: 10.3390/jof10050360 (PMC11122302; doi:10.3390/jof10050360)
Supplement: Supplementary file 1 [file jof-10-00360-s001.zip › supplementary_figures.pdf]

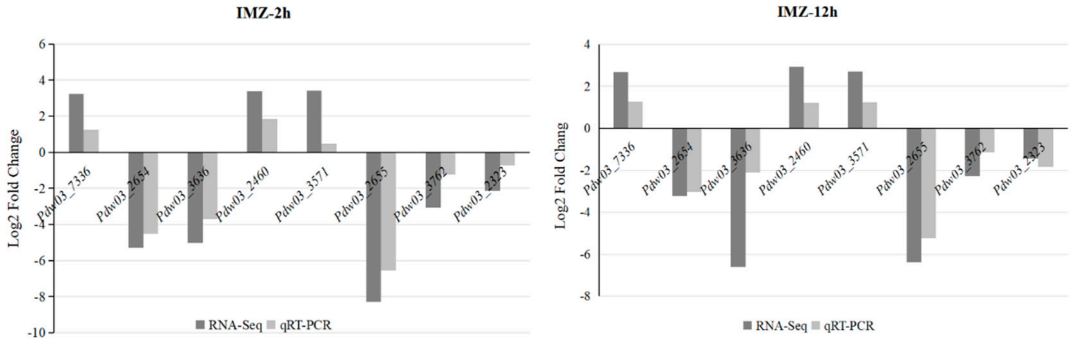

Fig. S1. qRT-PCR validation of the RNA-Seq data.

```
WT               AAAGAATTTATGTTTGGTATGCTTACGC-TAATGGCGTTTATTTTAGGTCGGCGACGCAA   355
ΔPdw03_3762-1    AAAGAATTTATGTTTGGTATGCTTACGCCTAATGGCGTTTATTTTAGGTCGGCGACGCAA   356
ΔPdw03_3762-13   AAAGAATTTATGTTTGGTATGCTTACGC--AATGGCGTTTATTTTAGGTCGGCGACGCAA   354

WT               GCGTACTCTTCCCCGGCCGACCAGCCTCTTGGCCGGCAGTGCTCCCCCCGGCCCCCAATC   178
ΔPdw03_3571-10   GCGTACTCTTCCCCGGCCGACCAGCCTCTTGGCCGGCAGTGCT-CCCCCGGCCCCCAATC   177

WT               CACCTCACCCTCCCAACCTGTCAGCCCC-G------------------------------   269
ΔPdw03_2654-5    CACCTCACCCTCCCAACCTGTCAGCCCC-GTCCCGAGCAAAAAGGATTGGAGGAGGAGGAG  300
ΔPdw03_2654-6    CACCTCACCCTCCCAACCTGTCAGCCCCCG------------------------------   270

WT               -------------------------------GGCGGGCAAGGCATCTCGAACGAAACCGC   298
ΔPdw03_2654-5    GAAGGTGAGAGTGAGACAAAGAGCGAAATAGGGCGGGCAAGGCATCTCGAACGAAACCGC   360
ΔPdw03_2654-6    -------------------------------GGCGGGCAAGGCATCTCGAACGAAACCGC   299
```

Fig. S2. Sequence alignment of gene locus in the selected mutants. Protospacers were marked in red. Protospacer-adjacent motifs (PAM) were underlined. Nucleotide alterations are highlighted in yellow.

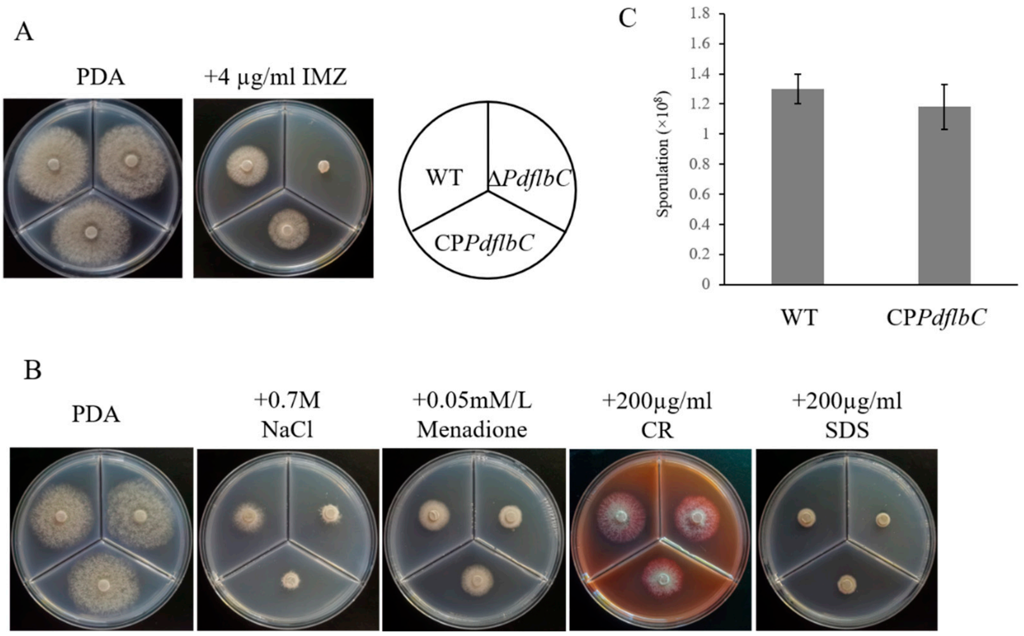

Fig. S3. Phenotypes of the complemented strain CP*PdflbC*. A) Sensitivity of the wild type, the gene

deletion mutant Δ*PdflbC* and the complemented strain CP*PdflbC* to IMZ. B) Sensitivity of the wild type, the gene deletion mutant Δ*PdflbC* and the complemented strain CP*PdflbC* to environmental stresses. C) Sporulation of the wild type and the complemented strain CP*PdflbC*.
