# Supplementary material for: Transcriptome Analysis Reveals Potential Regulators of DMI Fungicide Resistance in the Citrus Postharvest Pathogen Penicillium digitatum
Source: J Fungi (Basel). 2024 May 18;10(5):360. doi: 10.3390/jof10050360 (PMC11122302; doi:10.3390/jof10050360)
Supplement: Supplementary file 1 [file jof-10-00360-s001.zip › supplementry_tables.pdf]

Table S1. Primers used in this study.

Primer name	Sequencing (5'-3')	Relevant characteristics
pFC1	TACTGGGCCCGGGAAGATCTGCGTAAGCTCCCTAATTGGC	gRNA construction
pCF2	ACAGCTATGACCATGAGATCTGAGCCAAGAGCGGATTCCTC	
pFC1-g3571-R	AGCTTACTCGTTTCGTCCTCACGGACTCATCAGGTTGGCCGGTGATGTCTGCTCAAGCG	gRNA construction
pCF2-g3571-F	AGTAAGCTCGTCGTTGGCCGGCAGTGCTCCCCCGTTTTAGAGCTAGAAATAGCAAGTTAAA	
pFC1-g3762-R	AGCTTACTCGTTTCGTCCTCACGGACTCATCAGGTTTGGCGGTGATGTCTGCTCAAGCG	gRNA construction
pCF2-g3762 -F	AGTAAGCTCGTCGTTTGGTATGCTTACGCTAAGTTTTAGAGCTAGAAATAGCAAGTTAAA	
pFC1-g2654-R	AGCTTACTCGTTTCGTCCTCACGGACTCATCAGgCCCAACGGTGATGTCTGCTCAAGCG	gRNA construction
pCF2-g2654 -F	AGTAAGCTCGTCgCCCAACCTGTCAGCCCCGGGGTTTTAGAGCTAGAAATAGCAAGTTAAA	
g3571-JD-F	TCGGCTGATTCCTACGGT	transformants identification
g3571-JD-R	CAAGGCACAGGGAGAACG	
g3762-JD-F	TCCAACGACTGCGGACTT	transformants identification
g3762-JD-R	TGGCGGCTCACGAGATAG	
g2654-JD-F	GAGTAGTGAAACCGGAAACG	transformants identification
g2654-JD-R	GCACCATCTGCGACTGAC	
3530-Q-F:	TGTTAGCACATCGCTTCCTG	Amplify the *Pdw03_3530* gene in qRT-PCR assays
3530-Q-R:	GCCATACTCATACTCGCATCAC	
3762-Q-F:	CAGGATGAGAAACGGGAACA	Amplify the *Pdw03_3762* gene in qRT-PCR assays

3571-Q-F	CCAACACCCAGAAGAAGCACA	Amplify the *Pdw03_3571*gene in qRT-PCR assays
3571-Q-R	TCGTCATCGTCACCAGAGCC	
3636-Q-F	CTCGCTTCCTCCCTACAA	Amplify the Pdw03_3636 gene in qRT-PCR assays
3636-Q-R	AGGTCGGCTACAGTCTCG	
2655-Q-F	CTTGAGGCAAATCTCCCA	Amplify the *Pdw03_2655* gene in qRT-PCR assays
2655-Q-R	TGCTTTCATCGGTCCACT	
2654-Q-F	ATCATCAAATCAACCACCAA	Amplify the *Pdw03_2654* gene in qRT-PCR assays
2654-Q-R	CCACTATTAGGCTCGTAAGG	
7336-Q-F	ATTCAGCATTCGCTCTGTAGTCT	Amplify the *Pdw03_7336* gene in qRT-PCR assays
7336-Q-R	CCATTTCGGTTTCATCCATC	
2323-Q-F	GGAGGGTGGTGGATGTGA	Amplify the *Pdw03_2323* gene in qRT-PCR assays
2323-Q-R	CGGGACTGTGGGAGTTGT	
2460-Q-F：	TCTTCCTCTTCGCCTTCTCC	Amplify the *Pdw03_2460* gene in qRT-PCR assays
2460-Q-R：	GCCTTGTTGTAATAGCCGTTGT	
CYP51A-F	GTCGTCATACCTCGAACTCCC	Amplify the *CYP51A* gene in qRT-PCR assays
CYP51B-R	CGGATTGGCCTTTGAAGCTG	
CYP51B-F	CACAGAAGAAGTTGACCGAGAC	Amplify the *CYP51B* gene in qRT-PCR assays
CYP51B-R	GGATGACGACGAGTGATGG	
Actin-F	TCCACTACTGCCGAGCGTGAAAT	Amplify the Actin gene in qRT-PCR assays
Actin-R	CCGCCAGACTCAAGACCAAGAAC	
4069-F：	ACGTATGGCTACATCACTGACTC	Amplify the ERG24 gene in qRT-PCR assays
4069-R：	CAAACTACATCGCCGAAAGA	
7095-F：	AGTCCTTCGCCGATGCTC	Amplify the ERG1 gene in qRT-PCR assays
7095-R：	ATGCCTCCACCCGTGAGA	
386-F：	AGGCTGTTTGGTCTGTTTGT	Amplify the ERG3A gene in qRT-PCR assays
386-R：	GTGTCGTAGAGTTTGGCGTAT	

2864-F：	TACCTGGCATTACTTCTCCC	Amplify the ERG25 gene in qRT-PCR assays
2864-R：	GAAGCCCAAGATCATCACTT	
3754-F：	TCGTGAGGTATGGCAAAGCG	Amplify the ERG6 gene in qRT-PCR assays
3754-R：	AGCGGCAGAAATGGAAGGAG	
2561-F：	GAACCTGAAAGTCCGCAACG	Amplify the ERG5 gene in qRT-PCR assays
2561-R：	GGGTAAGCCTCCTCATCGTG	
6706-F：	AGGGTGAGGAGTGCATTGTT	Amplify the ERG4A gene in qRT-PCR assays
6706-R：	TACTCAGCAGGGTCGTGGTT	
2117-F：	TGGTGTCCCTATGTCTTACTG	Amplify the ERG4B gene in qRT-PCR assays
2117-R：	CTTCTGGCTGTTTGCTGTG	
7067-F：	TTCACAGATAGCGGGATTTA	Amplify the ERG3B gene in qRT-PCR assays
7067-R：	GGTAGACATGGTAGGGCAGA	

Table S2. Statistics of sequencing data.

SampleID	Total Reads	BaseSum	GC(%)	Q20(%)	Q30(%)	Mapped Reads	Uniq Mapped Reads	Multiple Map Reads
CK-1	53616314	8024495430	53.84	97.77	89.25	46,590,024 (86.90%)	46,332,920 (86.42%)	257,104 (0.48%)
CK-2	57572066	8616132164	53.84	98.08	90.64	50,621,319 (87.93%)	50,372,154 (87.49%)	249,165 (0.43%)
CK-3	59750474	8940526268	53.71	98.09	90.72	52,605,895 (88.04%)	52,300,386 (87.53%)	305,509 (0.51%)
IMZ12h-1	41677974	6237134752	53.63	98.78	93.08	39,124,888 (93.87%)	38,953,257 (93.46%)	171,631 (0.41%)
IMZ12h-2	47531050	7112072644	53.64	98	90.3	41,953,633 (88.27%)	41,709,160 (87.75%)	244,473 (0.51%)
IMZ12h-3	52769732	7897659212	53.8	98.04	90.51	46,537,883 (88.19%)	46,324,455 (87.79%)	213,428 (0.40%)
IMZ2h-1	49636364	7426564082	53.56	98.17	91.03	44,481,475 (89.61%)	44,305,115 (89.26%)	176,360 (0.36%)

IMZ2h-2	58121086	8698518980	53.58	98.02	90.4	51,612,383 (88.80%)	51,380,291 (88.40%)	232,092 (0.40%)
IMZ2h-3	51602940	7724795970	53.9	97.92	89.92	46,099,477 (89.33%)	45,921,451 (88.99%)	178,026 (0.34%)

Table S3. Differentially expressed transcription factors after IMZ treatment.

geneID	Annotation	2h-Log2FC	12h-Log2FC	TF
Pdw03_2654	Basic-leucine zipper (bZIP) transcription factor	-5.277533055	-3.222305046	bZIP_1
Pdw03_7664	C6 transcription factor, putative	-3.954842386	-2.989891018	Zn_clus
Pdw03_7446	PHD transcription factor (Rum1), putative	-3.290720486	-3.151349521	ARID
Pdw03_5422	Fungal transcriptional regulatory protein, N-terminal	-4.537605924	-1.496805996	Zn_clus
Pdw03_1620	Pyruvate/Phosphoenolpyruvate kinase	-2.314303153	-3.683901617	Fungal_trans
Pdw03_4243	Winged helix-turn-helix transcription repressor DNA-binding	-3.072669681	-2.491612848	Fork_head
Pdw03_4194	Zinc finger, C2H2	-2.051737923	-3.328531995	zf-C2H2
Pdw03_3762	Fungal transcriptional regulatory protein, N-terminal	-3.082146853	-2.285568712	Zn_clus
Pdw03_3206	Aflatoxin biosynthesis regulatory protein	-2.863532581	-2.131716068	Zn_clus
Pdw03_5090	Protease inhibitor I4, serpin	-2.24718469	-2.55681538	Zn_clus
Pdw03_4703	C2H2 transcription factor (Rpn4), putative	-2.924428711	-1.521661744	zf-C2H2
Pdw03_8176	GATA factor SREP	-2.05753796	-2.032890953	GATA
Pdw03_2365	Zn(2)-C6 fungal-type DNA-binding domain	-2.331173497	-1.752696665	Zn_clus
Pdw03_3389	Basic-leucine zipper (bZIP) transcription factor	-1.862816276	-2.094483706	bZIP_1
Pdw03_7768	Uracil-DNA glycosylase	-1.760556056	-1.921033059	Fungal_trans
Pdw03_3922	pH-response transcription factor pacC/RIM101	-1.281121113	-2.317608671	zf-C2H2
Pdw03_8795	Fungal transcriptional regulatory protein, N-terminal	-1.289465478	-2.0743941	Zn_clus
Pdw03_6801	Homeobox transcription factor (RfeB), putative	-1.082151854	-2.255542957	Homeobox
Pdw03_1622	Pyruvate/Phosphoenolpyruvate kinase	-1.304302023	-1.989166656	Zn_clus
Pdw03_4334	Fungal specific transcription factor, putative	-1.674763231	-1.568344224	Zn_clus
Pdw03_8415	Transcriptional regulator	-2.122343752	-1.058645786	zf-C2H2

Pdw03_4859	C6 transcription factor, putative	-1.739536797	-1.36586337	Zn_clus
Pdw03_2353	Fungal transcriptional regulatory protein, N-terminal	-1.656515291	-1.435342234	Zn_clus
Pdw03_5802	Zinc finger, C2H2-like	-1.76768398	-1.095455284	zf-C2H2
Pdw03_5521	Fungal transcriptional regulatory protein, N-terminal	-1.472516178	-1.371394588	Zn_clus
Pdw03_1773	Non-histone chromosomal protein 6	-1.160980152	-1.597176957	HMG_box
Pdw03_7815	Nitrogen assimilation transcription factor nit-4	-1.370932517	-1.316195242	Zn_clus
Pdw03_7397	C6 transcription factor, putative	-1.410439571	-1.001334238	Zn_clus
Pdw03_7117	Helix-loop-helix DNA-binding	-1.084308743	-1.1776687	HLH
Pdw03_3834	C6 transcription factor OefC	-1.099214596	-1.006203653	Zn_clus
Pdw03_1121	Fungal transcriptional regulatory protein, N-terminal	1.006093879	1.081296982	Zn_clus
Pdw03_6778	Fungal transcriptional regulatory protein, N-terminal	1.240236789	1.01559008	Zn_clus
Pdw03_4388	BZIP transcription factor, putative	1.06478547	1.199523445	bZIP_1
Pdw03_4216	C6 transcription factor, putative	1.187094091	1.22706668	Zn_clus
Pdw03_1937	Winged helix-turn-helix transcription repressor DNA-binding	1.115770981	1.299985248	Fork_head
Pdw03_4483	Fungal transcriptional regulatory protein, N-terminal	1.324844271	1.15056483	Zn_clus
Pdw03_2083	Zn(2)-C6 fungal-type DNA-binding domain protein	1.089311174	1.408277236	Zn_clus
Pdw03_8240	MADS box transcription factor Mcm1	1.517325935	1.050142498	SRF-TF
Pdw03_5998	putative transcriptional regulatory protein	1.106514825	1.527346247	Zn_clus
Pdw03_4875	C6 transcription factor RosA-like, putative	1.346633966	1.32768232	Zn_clus
Pdw03_3221	Fungal transcriptional regulatory protein, N-terminal	1.569397568	1.257424451	Zn_clus
Pdw03_8372	Crotonase, core	1.655157074	1.179922712	Zn_clus
Pdw03_3819	C2H2 transcription factor, putative	1.133373819	1.800863227	zf-C2H2
Pdw03_3511	C6 transcription factor, putative	1.071951431	1.904047227	Fungal_trans
Pdw03_2703	Zn(2)-C6 fungal-type DNA-binding domain	1.601193745	1.436383037	Zn_clus
Pdw03_2910	Zn(II)2Cys6 transcription factor	1.405417185	1.65099444	Zn_clus
Pdw03_5143	Fungal transcriptional regulatory protein, N-terminal	1.079126964	2.041288803	Zn_clus

Pdw03_2909	Zn(II)2Cys6 transcription factor	1.651838646	1.502391416	Fungal_trans
Pdw03_8375	Zn(II)2Cys6 transcription factor	1.548544733	1.731357513	Fungal_trans_2
Pdw03_6876	Fungal transcriptional regulatory protein, N-terminal	1.676364772	1.665870421	Zn_clus
Pdw03_2841	Ubiquitin-conjugating enzyme E2 6	1.436135989	1.923939917	Fungal_trans_2
Pdw03_8470	Fungal transcriptional regulatory protein, N-terminal	1.656096208	1.721453297	Zn_clus
Pdw03_8563	NDT80 / PhoG like DNA-binding family protein	1.133101834	2.251507565	NDT80_PhoG
Pdw03_8042	Fungal transcriptional regulatory protein, N-terminal	1.510786239	1.892390311	zf-C2H2
Pdw03_6475	C6 transcription factor (PrnA), putative	1.879398891	1.546664826	Zn_clus
Pdw03_8511	Nitrogen assimilation transcription factor nirA	2.322853838	1.108029983	Zn_clus
Pdw03_8248	Zinc finger, C2H2	1.725170568	1.722909652	zf-C2H2
Pdw03_4853	Fungal transcriptional regulatory protein, N-terminal	1.755883389	1.717378541	Zn_clus
Pdw03_6734	C6 transcription factor, putative	1.625373576	1.86077108	Zn_clus
Pdw03_8324	Zn(2)-C6 fungal-type DNA-binding domain	2.284554098	1.40701085	Fungal_trans_2
Pdw03_4958	C6 transcription factor, putative	1.570917316	2.1493182	Fungal_trans
Pdw03_3512	C6 transcription factor SndA, putative	1.901216561	1.850359802	Zn_clus
Pdw03_4894	Helix-loop-helix DNA-binding	2.299159573	1.483272197	HLH
Pdw03_8323	Fungal transcriptional regulatory protein, N-terminal	2.1543773	1.687916376	Zn_clus
Pdw03_8357	C6 transcription factor, putative	2.383815359	1.492877734	Zn_clus
Pdw03_5577	Fungal transcriptional regulatory protein, N-terminal	2.282020274	1.616108101	Fungal_trans
Pdw03_2416	Zinc finger, C2H2	1.50293679	2.515142853	zf-C2H2
Pdw03_8169	Fungal transcriptional regulatory protein, N-terminal	2.086893538	2.001197701	Zn_clus
Pdw03_1634	C6 transcription factor, putative	2.199539519	1.923919637	Zn_clus
Pdw03_6398	Fungal specific transcription factor, putative	1.785094658	2.351014078	Zn_clus
Pdw03_7652	Major facilitator superfamily domain, general substrate transporter	2.219900272	2.17180482	zf-C2H2
Pdw03_4980	C6 transcription factor, putative	2.816385349	1.607752323	Zn_clus
Pdw03_2719	Aflatoxin biosynthesis regulatory protein	2.179193277	2.245882288	Zn_clus

Pdw03_2541	Fungal transcriptional regulatory protein, N-terminal	2.572974818	2.034802517	Fungal_trans_2
Pdw03_4048	DNA binding HTH domain, Psq-type	1.964900208	2.687450191	CENP-B_N
Pdw03_7357	Blue (type 1) copper domain	2.54780061	2.132054444	Fungal_trans
Pdw03_2337	Fungal transcriptional regulatory protein, N-terminal	2.632414376	2.076137225	Zn_clus
Pdw03_7674	C6 transcription factor, putative	1.673862415	3.063508157	Fungal_trans
Pdw03_4267	C6 sexual development transcription factor NosA	1.875303027	3.007631528	Zn_clus
Pdw03_3530	Zinc finger, NHR/GATA-type	3.380534425	2.051652253	GATA
Pdw03_6775	Homeobox transcription factor, putative	2.190338624	3.45471108	Homeobox
Pdw03_7068	Fungal transcriptional regulatory protein, N-terminal	1.951341751	3.820371396	Fungal_trans
Pdw03_7336	Zinc finger, C2H2-type/integrase, DNA-binding	3.229268341	2.691057417	zf-C2H2
Pdw03_3571	C2H2 conidiation transcription factor FlbC	3.418330825	2.717238277	zf-C2H2
